# A Kernel-Based Calibration Algorithm for Chromatic Confocal Line Sensors

**DOI:** 10.3390/s24206649

**Published:** 2024-10-15

**Authors:** Ming Qin, Xiao Xiong, Enqiao Xiao, Min Xia, Yimeng Gao, Hucheng Xie, Hui Luo, Wenhao Zhao

**Affiliations:** 1 School of Optical and Electronic Information, Huazhong University of Science and Technology, Wuhan 430074, China; qinming@wuhanjingce.com (M.Q.);; 2 Wuhan Jingce Electronic Group Co., Ltd., Wuhan 430074, China

**Keywords:** chromatic confocal line sensors, wavelength calibration, kernel method, groove fitting

## Abstract

In chromatic confocal line sensors, calibration is usually divided into peak extraction and wavelength calibration. In previous research, the focus was mainly on peak extraction. In this paper, a kernel-based algorithm is proposed to deal with wavelength calibration, which corresponds to the mapping relationship between peaks (i.e., the wavelengths) in image space and profiles in physical space. The primary component of the mapping function is depicted using polynomial basis functions, which are distinguished along various dispersion axes. Considering the unknown distortions resulting from field curvature, sensor fabrication and assembly, and even the inherent complexity of dispersion, a typical kernel trick-based nonparametric function element is introduced here, predicated on the notion that similar processes conducted on the same sensor yield comparable distortions.To ascertain the performance with and without the kernel trick, we carried out wavelength calibration and groove fitting on a standard groove sample processed via glass grinding and with a reference depth of 66.14 μm. The experimental results show that depths calculated by the kernel-based calibration algorithm have higher accuracy and lower uncertainty than those ascertained using the conventional polynomial algorithm. As such, this indicates that the proposed algorithm provides effective improvements.

## 1. Introduction

Based on the confocal principle [1,2,3], a series of point sensors, such as laser microscopy, confocal fluorescence microscopy, and chromatic confocal microscopy [4,5,6], have been developed. Due to their high precision and non-contact nature, confocal sensors are predominantly utilized in the field of 3D topography measurement. A motion device capable of at least 2D translation has to be employed in order to achieve full-field 3D scanning. In order to reduce the mechanical movement and shorten the measurement times, a chromatic confocal line sensor using a line focus and a spectrometer was produced by Ruprecht [7]. Their sensor enables measurement parallelization in the *X* direction (i.e., the lateral dimension). As a result, compared with traditional point sensors, this line sensor shows great potential for high-efficiency 3D measurements. With the increasing requirements of precision manufacturing, more advanced line sensors were proposed by Seppä et al. [8] and Hu et al. [9].

The key to measuring using chromatic confocal line sensors lies in calibrating from peaks in image space to profiles in physical space. This is similar to chromatic confocal point sensors [10,11,12,13], which are usually divided into peak extraction and wavelength calibration. Much research has been carried out on the former. Combined with data preprocessing, the Gaussian fitting method was chosen as the best by both Luo et al. [14] and Li et al. [15]. According to Monte Carlo simulations, optical aberration influences, random noise, and sample surface height on peak extraction were evaluated by Chen et al. [16,17]. In order to balance calculation efficiency and extraction accuracy, a mean-shift vector-based peak extraction method was put forward by Lu et al. [18]. Their method had an accuracy equal to that achieved with the Gaussian fitting method, while its efficiency was improved by over 70 times. Similarly, Liu et al. [19] proposed an efficient and accurate peak extraction method using shifting, difference, linear fitting, zero point, and peak abscissa calculations for real-time dynamic online detection. However, in the past, few studies have been conducted on wavelength calibration. In most cases, the polynomial fitting method is chosen as the default in establishing wavelength–displacement relationships, such as linear approximation [12,13,14], quadratic approximation [20] and cubic approximation [8,9]. For further improved measurement accuracy, wavelength–displacement relationship corrections have been investigated via temperature effects [21] and colored specimens [22].

In this paper, we propose a kernel-based algorithm to deal with wavelength calibration. Under the assumption of similar distortions on different dispersion axes, the kernel trick-based wavelength–displacement relationships are obtained. After transforming in the *Z* direction, a parametric model is built to acquire the lateral resolution, thus enabling transformation in the *X* direction. Peaks in image space are converted to profiles in physical space after these two kinds of transformations. Finally, a groove fitting model based on the least squares method is deduced for reliable groove depth calculations. In this way, we can evaluate the proposed algorithm against the conventional polynomial algorithm.

## 2. Methods

### 2.1. Coordinate Systems

**Figure 1:**
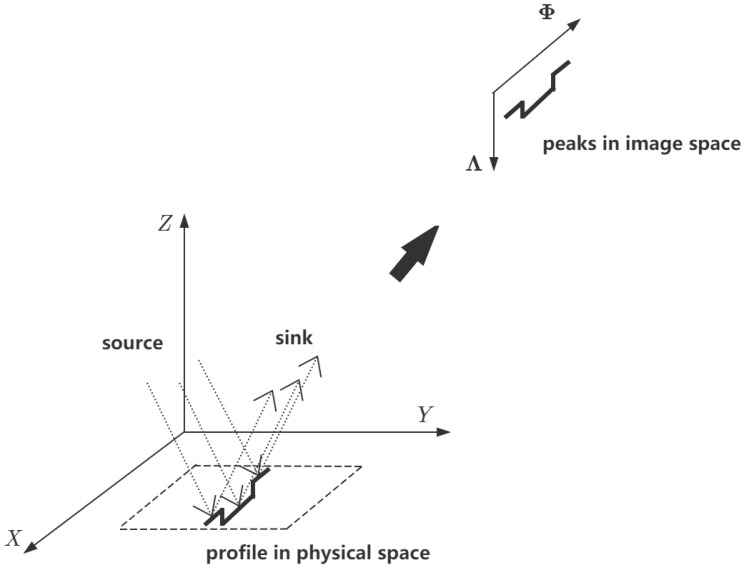
Coordinate systems in image and physical spaces. Peaks and profiles are distributed in image and physical spaces, respectively.

In chromatic confocal point sensors, wavelength calibration only refers to the transformation in the *Z* direction. Here, the transformation in the *X* direction is also involved as a result of an additional lateral dimension along the profile line. As shown in Figure 1, the lower left part and the upper right part represent the physical and the image spaces, respectively. The depth and lateral dimensions coincide with the *Z* and *X* axes, respectively. The coordinates Φ (variable ϕ) and Λ (variable λ) in the image space map to the coordinates *X* (variable *x*) and *Z* (variable *z*).

**Figure 2:**
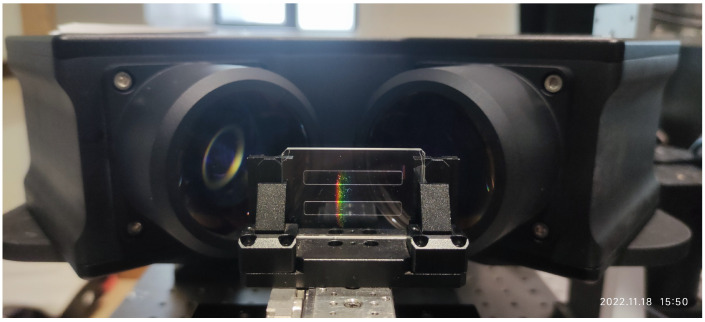
Measurement case in which a standard groove sample is fixed on a sliding platform, which is capable of translation in the *Z* direction.

Before wavelength calibration, peaks in the image space need to be extracted from the raw images. For this paper, all the raw images were captured using an chromatic confocal line sensor (LSCF1000, Jingce Electronic Group Co., Ltd., Wuhan, China), which our team developed over the past few years [23]. Figure 2 shows a measurement case with the sensor in a working state.

**Figure 3:**
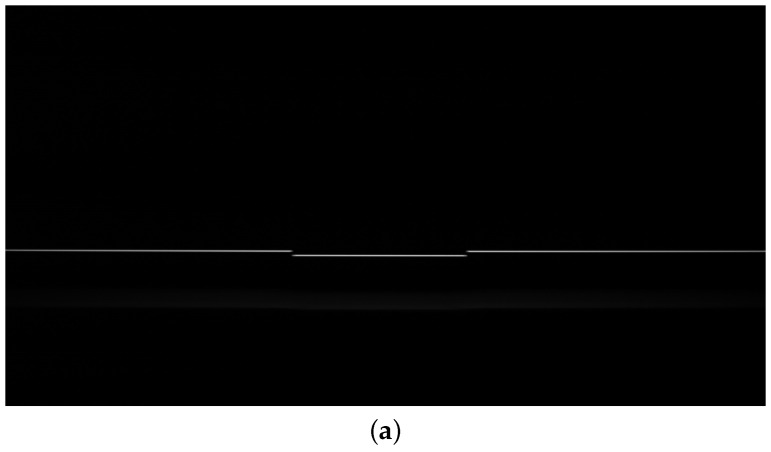

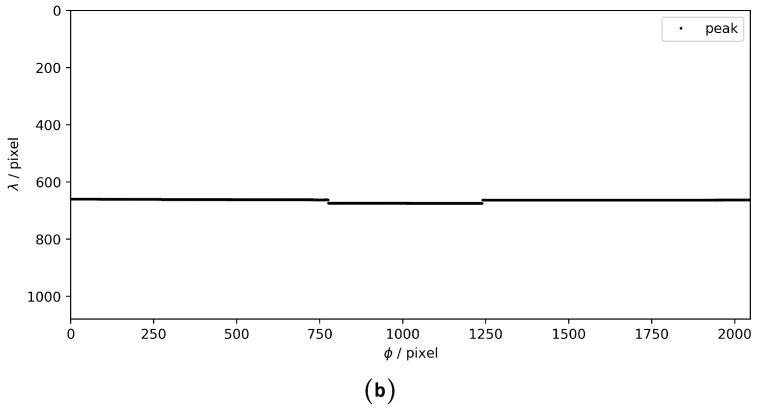
A raw image of a standard groove sample from the LSCF1000 and the corresponding peak extraction result using the centroid method. (**a**) A raw image of a standard groove sample. (**b**) Peak extraction on the raw image.

Given a set of appropriate exposure parameters, the sensor produces a series of raw images, the details of which are shown in Figure 3. The raw image (Figure 3a) was captured from a standard groove sample with a reference depth of 66.14 μm. We adapted the centroid method, which is still widely used [24,25], to extract the corresponding peak data (Figure 3b). Each column is correlated with a separate wavelength–displacement relationship.

### 2.2. Transformation in *Z* Direction

Based on the regular design of axial dispersion for chromatic confocal systems, the main component of the wavelength–displacement relationship within the measurement range behaves as a linear function [26], and the non-linear component is weak. Here, the wavelength–displacement relationships on different columns in the image space are separate. Owing to the similar operations on the same chromatic confocal line sensor, such as field curvature, sensor fabrication and assembly, and the inherent complexity of dispersion, it is assumed that similar distortions happen to the spectra of the line source. Hence, the wavelength–displacement relationship on the ϕ-th column can be written as

(1) $$z=f_{\phi }\left(\lambda \right)+g\left(\lambda \right),$$

where λ and *z* represent the wavelength in the image space and the displacement in the physical space, respectively, and functions $f_{\phi }$ and *g* are the separate near-linear component and the common non-linear component, respectively.

Without generality loss, $f_{\phi }$ and *g* can be obtained using the following formulas:

(2) $$f_{\phi }\left(\lambda \right)=W_{\phi }^{T}P\left(\lambda \right),$$

(3) $$g\left(\lambda \right)=\alpha^{T}K\left(A^{T},\lambda \right),$$

where *P* is a feature matrix built on the polynomial basis, *K* is a kernel matrix built on the kernel trick, and *A* is a hyper-parameter composed of different wavelengths.

According to Equations (2) and (3), Equation (1) is converted to

(4) $$z=W_{\phi }^{T}P\left(\lambda \right)+\alpha^{T}K\left(A^{T},\lambda \right).$$

Thus, function *z* is a linear system with regard to the undetermined coefficients, $W_{\phi }$ and α.

**Figure 4:**
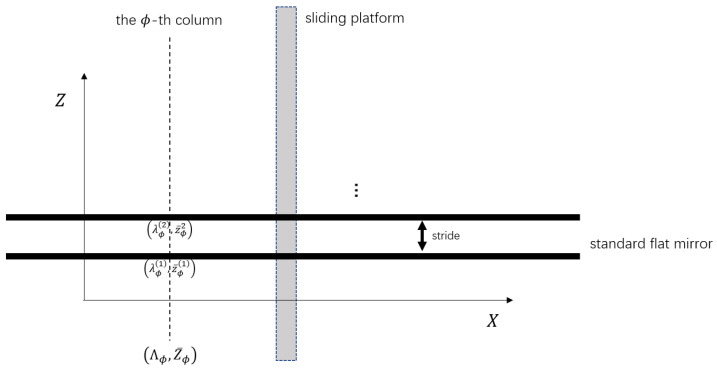
Data collection for transformation in the *Z* direction. The standard flat mirror parallel to the XY plane is fixed on a sliding platform capable of translation in the *Z* direction. The wavelength λ on the ϕ-th column refers to the peak extracted from the image space, and the corresponding displacement $\bar{z}$ is obtained from the sliding platform.

In order to ascertain the wavelength–displacement relationship on each column, datasets $\left\{\left(\Lambda_{\phi },{\bar{Z}}_{\phi }\right)\right\}\left(\phi =1,\cdots ,n\right)$ are collected from a standard flat-mirror sample parallel to the XY plane moving along the *Z* direction. Details in this regard are given in Figure 4. Combining the least squares method and $L_{2}$ regularization yields the following convex problem:

(5) $$\begin{array}{cc} \min\; & \frac{1}{2}\sum_{\phi }∥\xi_{\phi }{∥}_{2}^{2}+\frac{c}{2}{∥\alpha ∥}_{2}^{2}\;\\ s.t.\; & {\bar{Z}}_{\phi }-\left(W_{\phi }^{T}P\left(\Lambda_{\phi }\right)+\alpha^{T}K\left(A^{T},\Lambda_{\phi }\right)\right)=\xi_{\phi },\;\phi =1,\cdots ,n, \end{array}$$

where *c* is the weight of the regularization term, *n* is the total number of columns, and ξ represents the error between the standard value read from the sliding platform and the predicted value given by the kernel method. After choosing the polynomial basis and the kernel function, we can solve the above optimization problem in Equation (5). Then, transformation in the *Z* direction can be performed using Equation (4) once the column index ϕ and the corresponding peak wavelength λ are given.

### 2.3. Transformation in *X* Direction

As a result of the line source, the lateral relationship between *x* and ϕ can usually be considered linear scaling, i.e.,

(6) x=rϕ,

where ϕ and *x* represent the column index in the image space and the lateral position in the physical space, and *r* is the lateral resolution. Hence,

(7) $$\frac{dz}{dx}=\frac{1}{r}\frac{dz}{d\phi },$$

where $\frac{dz}{dx}$ and $\frac{dz}{d\phi }$ represent *X*-tilt slopes in the physical and transitional spaces, respectively. Both slopes are correlated with a standard flat-mirror sample fixed on a rotation device whose *X*-tilt angle θ can be read directly.

However, it is hard to keep the *X*-axis of both the rotation device corresponding to θ=0 and the sensor’s coordinate system aligned. A constant bias *b* should be introduced as follows:

(8) $$\frac{dz}{dx}=\tan\left(\theta +b\right).$$

After transformation in the *Z* direction using Equation (4), the coordinates (ϕ,λ) in image space of a standard flat-mirror sample at different *X*-tilt angles are converted to (ϕ,z) in the transitional space. Therefore, different *X*-tilt slopes in the transitional space (i.e., *k*) can be computed from linear regression, i.e.,

(9) $$\frac{dz}{d\phi }=k.$$

According to Equations (7)–(9), slope *k* can be written as

(10) k=rtan(θ+b),

where *r* and *b* are undetermined, and θ and *k* are known.

**Figure 5:**
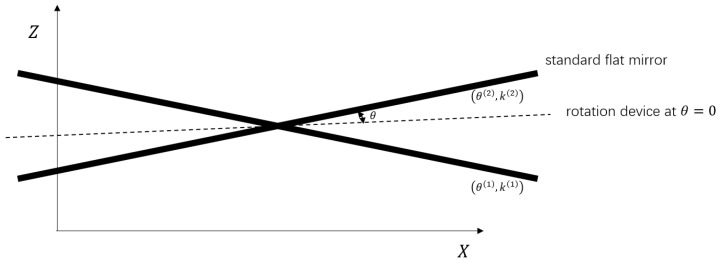
Data collection for transformation in the *X* direction. The standard flat mirror is fixed on a rotation device. The *X*-tilt angle θ of the standard flat mirror is obtained from the rotation device, and the corresponding slope *k* is calculated after transformation in the *Z* direction.

Assuming that *m* sets of data of a standard flat-mirror sample with different *X*-tilts are collected, the corresponding $\left\{\left(\theta^{\left(1\right)},k^{\left(1\right)}\right),\cdots ,\left(\theta^{\left(m\right)},k^{\left(m\right)}\right)\right\}$ can be acquired, the details of which are given in Figure 5. Via the least squares method, *r* and *b* can be computed as follows:

(11) $$r^{*},b^{*}=\underset{r,b}{\arg\min}\sum_{i}{\left(k^{\left(i\right)}-r\tan\left(\theta^{\left(i\right)}+b\right)\right)}^{2},$$

where $r^{*}$ and $b^{*}$ are the optimal solutions of lateral resolution and bias.

Finally, using Equation (6), transformation in the *X* direction can be performed once the column index ϕ is given.

## 3. Results

### 3.1. Groove Fitting

After transformations in the *Z* and *X* directions, peaks (ϕ,λ) in the image space are converted to profiles (x,z) in the physical space. The effectiveness of the kernel-based approach needs to be demonstrated by numerically comparing it with the conventional polynomial method. Here, calibrations of a standard groove sample are carried out to check the accuracy and the uncertainty of the proposed method.

**Figure 6:**
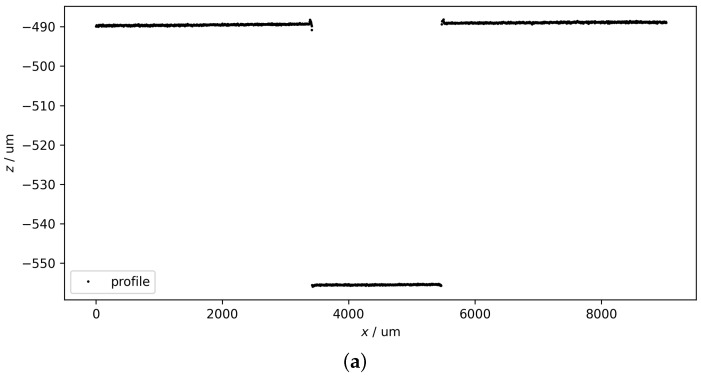

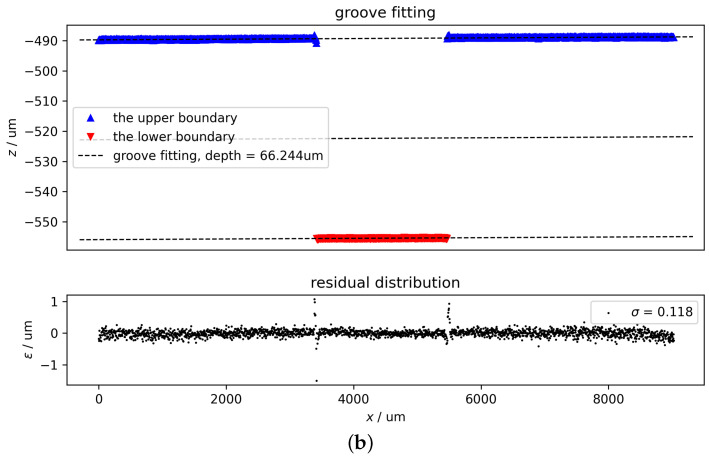
An example of groove depth calculation, which can be divided into two sequential subprocesses: (**a**) wavelength calibration and (**b**) groove fitting. The groove depth computed from the profile is about 66.24 μm.

Figure 6 shows an example of groove depth calculation consisting of two subprocesses: (a) wavelength calibration and (b) groove fitting. The original peak data are shown in Figure 3. The computation depth is about 66.24 μm in this case, which is close to the reference depth of 66.14 μm.

### 3.2. Depths at Different Heights

**Figure 7:**
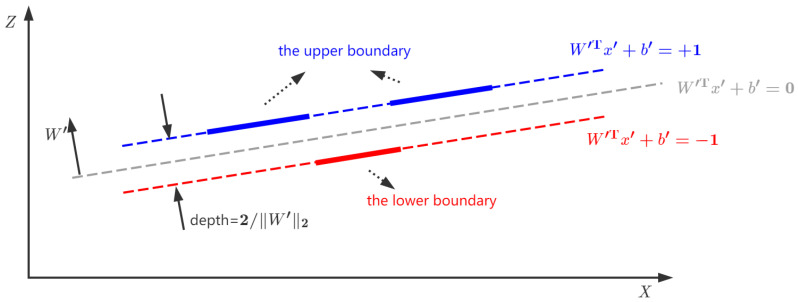
An ideal groove sample in physical space. The upper and lower boundaries coincide with two parallel straight dashed lines, the normal vector of which is $W^{\prime }$.

The profiles of a standard groove sample are composed of data points located on the upper and lower boundaries. As shown in Figure 7, these two boundaries coincide with two parallel straight dashed lines, the normal vector of which is assumed to be $W^{\prime }$. Without generality loss, the dashed straight line in the center can be written as

(12) $$W^{\prime T}x^{\prime }+b^{\prime }=0,$$

where $x^{\prime }$ represents the data point ${\left[x\;z\right]}^{T}$ in physical space. Accordingly, the other two dashed straight lines equidistant from the center line can be given by

(13) $$\begin{array}{cccc} W^{\prime T}x^{\prime }+b^{\prime } & =+1, & \; & \mathrm{for}\;\mathrm{the}\;\mathrm{upper}\;\mathrm{dashed}\;\mathrm{straight}\;\mathrm{line};\\ W^{\prime T}x^{\prime }+b^{\prime } & =-1, & \; & \mathrm{for}\;\mathrm{the}\;\mathrm{lower}\;\mathrm{dashed}\;\mathrm{straight}\;\mathrm{line}. \end{array}$$

The distance between these two dashed lines is $2/∥W^{\prime }{∥}_{2}$.

The upper and lower dashed straight lines are used for fitting data points on the upper and lower boundaries, respectively. The errors (i.e., $\varepsilon^{\prime }$ and $\varepsilon^{\prime \prime }$) of these data points on both boundaries can be introduced as follows:

(14) $$\begin{array}{cccc} W^{\prime T}x^{\prime }+b^{\prime } & =+1+\varepsilon^{\prime }, & \; & \mathrm{for}\;x^{\prime }\;\mathrm{on}\;\mathrm{the}\;\mathrm{upper}\;\mathrm{boundary},\\ W^{\prime T}x^{\prime }+b^{\prime } & =-1+\varepsilon^{\prime \prime }, & \; & \mathrm{for}\;x^{\prime }\;\mathrm{on}\;\mathrm{the}\;\mathrm{lower}\;\mathrm{boundary}. \end{array}$$

Moreover, another dimension called “label” is introduced here. On the upper and lower boundaries, $x^{\prime }$ is labeled as $\bar{y}=1$ and $\bar{y}=-1$, respectively. Hence, Formula (14) is equivalent to

(15) $$\begin{array}{cccc} \bar{y}\left(W^{\prime T}x^{\prime }+b^{\prime }\right) & =1+\varepsilon^{\prime }, & \; & \mathrm{for}\;x^{\prime }\;\mathrm{on}\;\mathrm{the}\;\mathrm{upper}\;\mathrm{boundary},\\ \bar{y}\left(W^{\prime T}x^{\prime }+b^{\prime }\right) & =1-\varepsilon^{\prime \prime }, & \; & \mathrm{for}\;x^{\prime }\;\mathrm{on}\;\mathrm{the}\;\mathrm{lower}\;\mathrm{boundary}. \end{array}$$

Meanwhile, there exists a minimum groove depth because of the profiles captured from different views of the measurement sensor. A constraint regarding the distance between these two boundary lines can be given by

(16) $$d=\frac{2}{∥W^{\prime }{∥}_{2}}\geq d_{\min},$$

where *d* and $d_{\min}$ represent the computation value and the minimum estimation of the groove depth, respectively.

According to the least squares method, a groove fitting model taking into account Equations (15) and (16) can be described by the following convex problem:

(17) $$\begin{array}{ll} \min\; & \frac{1}{2}{∥\varepsilon ∥}_{2}^{2}\;\\ s.t.\; & D[A^{\prime T}W^{\prime }+1b^{\prime }]=1+\varepsilon \;\\ \; & ∥W^{\prime }{∥}_{2}^{2}\leq c, \end{array}$$

where $c=4/d_{\min}^{2}$. A small positive value (e.g., $1\times 10^{-6}$) could be assigned to $d_{\min}$ if the minimum estimation of the groove depth is unknown. Assuming that the number of data points of a groove profile is *l*, $A^{\prime }$ and *D* are as follows:

$$A^{\prime }=\left[\begin{array}{cccc} x^{\left(1\right)} & x^{\left(2\right)} & \cdots & x^{\left(l\right)}\\ z^{\left(1\right)} & z^{\left(2\right)} & \cdots & z^{\left(l\right)} \end{array}\right]\;\;D=\mathrm{diag}\left(\left[\begin{array}{cccc} {\bar{y}}^{\left(1\right)} & {\bar{y}}^{\left(2\right)} & \cdots & {\bar{y}}^{\left(l\right)} \end{array}\right]\right).$$

After solving this convex problem in Equation (17), the groove depth *d* can be computed using Equation (16).

### 3.3. Depths Measurements

**Figure 8:**
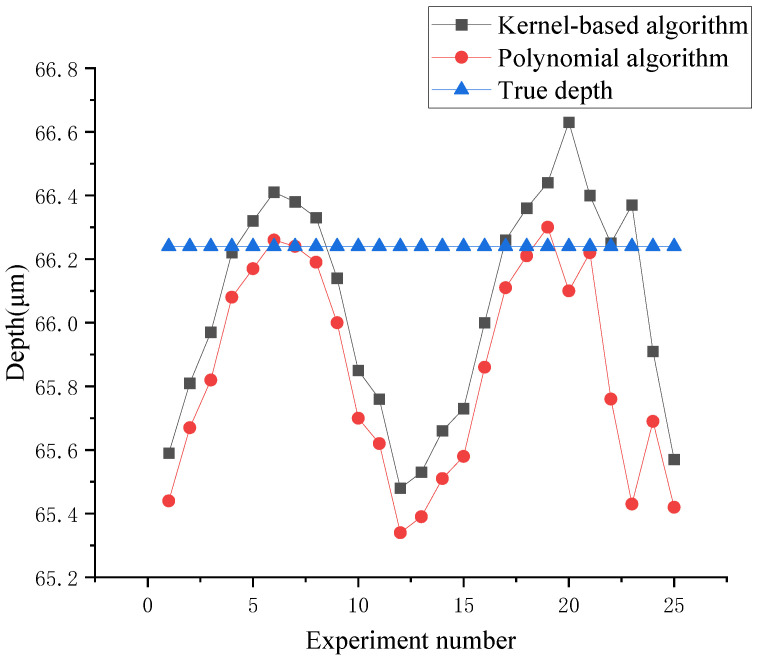
Comparison of deviation from true depth between polynomial and kernel-based algorithms.

In order to evaluate the performance of calibration algorithms in terms of measurement, the depths of the same groove sample were computed by the algorithms with and without the kernel trick, and the measurement experiment was repeated 25 times to evaluate the performance of the algorithm. The corresponding results are shown in Table 1 and the comparison of deviations from true depths with and without the kernel trick is shown in Figure 8. Thus, the average error and the standard deviation of the kernel-based algorithm are reduced by 69% and 24%, respectively, compared to the polynomial algorithm. The measurement accuracy was improved significantly by this calibration process. It can be concluded that depths have higher accuracy and lower uncertainty when using the kernel-based algorithm than the polynomial algorithm. This indicates that the kernel trick makes an effective improvement in terms of measurement.

## 4. Conclusions

In this paper, a kernel-based calibration algorithm for chromatic confocal line sensors was developed. The kernel trick was introduced to describe the common non-linear component of wavelength–displacement relationships on different columns. Strides were applied to data collection through translation in the *Z* direction and rotation at different *X*-tilts (these were 10 μm and 1°, respectively) in order to compute the transformation coefficients in wavelength calibration. After transformations in the *Z* and *X* directions, peaks in the image space were converted to profiles in the physical space. The depth measurements of the standard groove sample show that the proposed algorithm works with high accuracy and stability. The limitations of this work mainly lie in linear scaling in the *X* direction. In the future, we will explore how to avoid this linear limitation and figure out whether our algorithm, in turn, is helpful in evaluating the optical design of chromatic confocal line sensors.

## Figures and Tables

**Table 1:** Groove depths calculated by polynomial and kernel-based algorithms (μm).

	True Depth	Polynomial Algorithm		Kernel-Based Algorithm	
		**Depth**	**Error**	**Depth**	**Error**
1	66.14	65.44	−0.70	65.59	−0.55
2		65.67	−0.47	65.81	−0.33
3		65.82	−0.32	65.97	−0.17
4		66.08	−0.06	66.22	0.08
5		66.17	0.03	66.32	0.18
6		66.26	0.12	66.41	0.27
7		66.24	0.10	66.38	0.24
8		66.19	0.05	66.33	0.19
9		66.00	−0.14	66.14	0.00
10		65.70	−0.44	65.85	−0.29
11		65.62	−0.52	65.76	−0.38
12		65.34	−0.80	65.48	−0.66
13		65.39	−0.75	65.53	−0.61
14		65.51	−0.63	65.66	−0.48
15		65.58	−0.56	65.73	−0.41
16		65.86	−0.28	66.00	−0.14
17		66.11	−0.03	66.26	0.12
18		66.21	0.07	66.36	0.22
19		66.30	0.16	66.44	0.30
20		66.10	−0.04	66.63	0.49
21		65.22	0.08	66.40	0.26
22		65.76	−0.38	66.25	0.11
23		65.43	−0.71	66.37	0.23
24		65.69	−0.45	65.91	−0.23
25		65.42	−0.72	65.57	−0.57
Average error			−0.29		−0.09
Standard deviation			0.50		0.38

## Data Availability

The data presented in this study are available on request from the corresponding author.
